# Census timing alters stage duration distributions in matrix population models

**DOI:** 10.1002/ece3.5315

**Published:** 2019-07-09

**Authors:** Toshinori Okuyama

**Affiliations:** ^1^ Department of Entomology National Taiwan University Taipei Taiwan

**Keywords:** demography, elasticity, population growth, stage structure

## Abstract

Matrix population models are widely used to study the dynamics of stage‐structured populations. A census in these models is an event monitoring the number of individuals in each stage and occurs at discrete time intervals. The two most common methods used in building matrix population models are the prebreeding census and postbreeding census. Models using the prebreeding and postbreeding censuses assume that breeding occurs immediately before or immediately after the censuses, respectively. In some models such as age‐structured models, the results are identical regardless of the method used, rendering the choice of method a matter of preference. However, in stage‐structured models, where the duration of the first stage of life varies among newborns, a choice between the prebreeding and postbreeding censuses may result in different conclusions. This is attributed to the different first‐stage duration distributions assumed by the two methods. This study investigated the difference emerging in the structures of these models and its consequence on conclusions of eigenvalue and elasticity analyses using two‐stage models. Considerations required in choosing a modeling method are also discussed.

## 1 INTRODUCTION

The demographic rates (e.g., survival and reproduction rates) of an individual change over the course of his/her life. Survivorship curves indicate that many species do not have a constant survival rate over their lifetime (Gibbons & Semlitsch, 1982; Pinder, Wiener, & Smith, 1978; Plough, Shin, & Hedgecock, 2016; Schaal & Leverich, 1982). Similarly, in species with distinct life stages (e.g., insects with complete metamorphosis), stage‐specific demographic rates may be imposed through stage‐specific species interactions, such as with egg parasitoids (Kivan & Kilic, 2006; Pilkington & Hoddle, 2006), larval parasitoids (Dannon, Tamò, Huis, & Dicke, 2010; Ris, Allemand, Fouillet, & Fleury, 2004), and pupal parasitoids (Wang, Kaçar, Biondi, & Daane, 2016; Wang & Liu, 2002). In addition, only reproductively mature individuals may reproduce and the reproductive rate may change over time after reaching reproductive maturity (Croft, Brent, Franks, & Cant, 2015; Nielsen, Hamilton, & Matadha, 2008). It is essential to account for such variations in demographic rates to accurately describe population dynamics. Matrix population models have been used in a wide variety of taxa such as plants (Shea & Kelly, 1998), arthropods (Bommarco, 2001), amphibians (Vonesh & De la Cruz, 2002), reptiles (Crouse, Crowder, & Caswell, 1987), fish (Morris, Shertzer, & Rice, 2011), birds (Hitchcock & Gratto‐Trevor, 1997), and mammals (Fujiwara & Caswell, 2002) to describe variation in demographic parameters among different life stages.

Matrix population models are discrete time models. When *n* distinct life stages are identified in a population, its dynamics may be described as

(1) x(t+1)=Ax(t)

where **x**(*t*) is a vector representing the number of individuals of each of the *n* stages at time *t* (e.g., the population is censused at time *t*), and **A** is an *n‐*by‐*n* matrix summarizing stage‐specific demographic processes (specific examples are provided below). In particular, the dominant eigenvalue of **A** (denoted by *λ*) is the finite rate of increase (Caswell, 2001; Otto & Day, 2007). In this study, the dominant eigenvalue and finite rate of increase are used interchangeably, and **A** is referred to as population matrix. Four types of events occur on the discrete time line: breeding, death, stage transition, and census. Because **x**(*t*) is the result of the census at time *t* set by the model formulation (Equation 1), models must specify how other demographic processes occur relative to the timing of the censuses.

Matrix models commonly assume birth pulse, in which breeding by all reproductively mature individuals occurs at the same time in each time step. Birth‐flow models where births occur continuously within a time step are also described (Caswell, 2001), but they are rarely used. Birth‐pulse models still have to determine the timing of breeding events relative to censuses. Two predominantly common formulations are the prebreeding census (Arnold, Brault, & Croxall, 2006; Stricker & Stiling, 2012) and postbreeding census (Bieber & Ruf, 2005; Crowder, Crouse, Heppell, & Martin, 1994). The prebreeding census assumes that a census is done immediately before a breeding pulse, whereas the postbreeding census assumes a census takes place immediately after a breeding pulse. In some models, the two approaches yield identical conclusions (Case, 2000). However, the choice between the prebreeding and postbreeding censuses may result in qualitatively different conclusions in stage‐structured population models.

The purpose of this study was to investigate an important difference between the prebreeding and postbreeding censuses emerging in stage‐structured matrix population models. Initially, prebreeding and postbreeding methods are reviewed to illustrate the difference. Subsequently, the effects of this difference in the typical matrix population model analyses (i.e., eigenvalue and elasticity analyses) are described. In addition, a common problem found in published studies related to this difference is briefly discussed.

## 2 MODELS

Although this study focused on stage‐structured models, the prebreeding and postbreeding censuses are initially described using age‐structured models. This is because the distinction between the two methods is clearer in age‐structured models than in stage‐structured models. In fact, the choice between the prebreeding and postbreeding censuses in age‐structured models is a matter of preference. Subsequently, why the same equivalence between the prebreeding and postbreeding censuses do not fold in stage‐structured models is described.

### 2.1 Age‐structured models

Age‐structured models are a special type of stage‐structured models in which the duration of each stage is constant for all individuals (i.e., each age group is a stage). Age‐specific demographic parameters are expressed as *σ_x_* describing the probability that an age‐*x* individual survives one time step, and the number of female offspring produced by an individual upon reaching her *x*th birthday, *b_x_*. In a hypothetical scenario supposing that all individuals die before reaching the age of 4 (age‐4), Equation (1) based on the postbreeding census is

(2) $$\left(\begin{array}{c} n_{0}\\ n_{1}\\ n_{2}\\ n_{3} \end{array}\right)\left(t+1\right)=\left(\begin{array}{cccc} \sigma_{0}b_{1} & \sigma_{1}b_{2} & \sigma_{2}b_{3} & 0\\ \sigma_{0} & 0 & 0 & 0\\ 0 & \sigma_{1} & 0 & 0\\ 0 & 0 & \sigma_{2} & 0 \end{array}\right)\left(\begin{array}{c} n_{0}\\ n_{1}\\ n_{2}\\ n_{3} \end{array}\right)\left(t\right)$$

where *n_x_*(*t*) is the number of age‐*x* individuals at time *t*. The corresponding model with the prebreeding census is

(3) $$\left(\begin{array}{c} n_{1}\\ n_{2}\\ n_{3} \end{array}\right)\left(t+1\right)=\left(\begin{array}{ccc} \sigma_{0}b_{1} & \sigma_{0}b_{2} & \sigma_{0}b_{3}\\ \sigma_{1} & 0 & 0\\ 0 & \sigma_{2} & 0 \end{array}\right)\left(\begin{array}{c} n_{1}\\ n_{2}\\ n_{3} \end{array}\right)\left(t\right).$$

The difference in the size of the matrices (Equations 2 and 3) illustrates the difference between the postbreeding and prebreeding censuses. In the postbreeding census, the youngest individuals at a census are those born immediately prior to the census and are identified as age‐0. On the other hand, in the prebreeding census, the youngest individuals at a census are those born at the previous breeding event that occurred one time step earlier, identified as age‐1. In other words, there are no age‐0 individuals in prebreeding census models at a census because they only occur between censuses, and thus, *n*_0_ does not appear in Equation (3).

**Figure 1:**
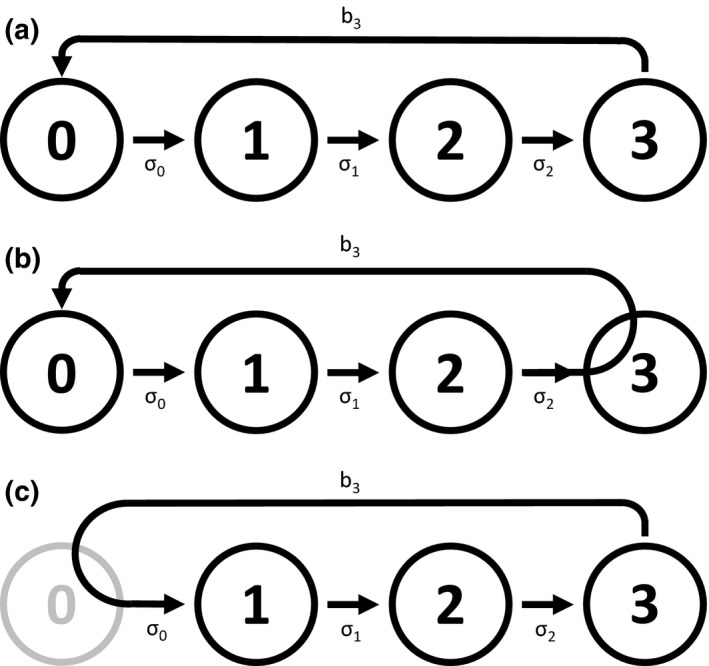
Life cycle diagrams for age‐structured models. Nodes represent age groups. Arrows represent reproduction and development that take place in one time step. (a) Life cycle diagram without being explicit about the timing of demographic processes and censuses. It is advised to interpret this diagram one demographic process at a time. (b) Life cycle diagram for the postbreeding census model (Equation 2 with *b*_1_ = *b*_2_ = 0). Arrows are modified according to the timing of demographic processes and censuses assumed in the model. For example, age‐2 individuals at the current census will become age‐3 and reproduce before the next census. Therefore, the arrow representing reproduction originates from age‐2 rather than age‐3. (c) Life cycle diagram for the prebreeding census model (Equation 3 with *b*_1_ = *b*_2_ = 0). There is no age‐0 individual at a census (represented by node 0 that is grayed out). The arrow representing reproduction originates from age‐3 and reaches age‐1 (by going through age‐0) because age‐3 individuals at the current census will immediately reproduce *b*_3_ offspring (i.e., age‐0), and all those age‐0 individuals will be age‐1 at the next census if they survive with probability σ_0_

Although the population matrices are different, both models describe the same demographic processes described in Figure 1a. In the diagram, each node represents an age group (e.g., node 0 represents age‐0). Arrows represent demographic processes (i.e., development and reproduction) that take place in one time step. For simplicity, the diagram assumes that only age‐3 can reproduce (i.e., *b*_1_ = 0 and *b*_2_ = 0 in Equations 2 and 3). The arrow originating age‐3 and pointing toward age‐0 represents this reproction. All other arrows indicate development (i.e., aging) in which when an age‐*x* individual survives with probability *σ_x_*, it becomes age‐(*x* + 1). It is important to note that Figure 1a arbitrarily describes demographics processes without being explicit about the timing of demographic processes and censuses. Therefore, it is only appropriate to interpret the diagram one demographic process at a time. If we want to model the processes described in Figure 1a in a biologically consistent manner, we must make assumptions about the relative timing of demographic processes and censuses (e.g., the postbreeding census or prebreeding census).

In the diagram representing the postbreeding census model (Figure 1b), each node represents respective age group at a census. One notable difference from Figure 1a is that the reproduction arrow (the arrow pointing toward age‐0) originates from age‐2 rather than age‐3. This is because age‐2 individuals become age‐3 and reproduce in one transition. There is no arrow originating from age‐3 (corresponding with column 4 in Equation (2) where all entries are 0).

In the prebreeding census model, there are no age‐0 individuals at a census, which is represented by node 0 that is grayed out (Figure 1c). The arrow representing reproduction goes through age‐0 and reaches age‐1. This is because newborns that appear immediately after a census and will become age‐1 at the next census.

Although the postbreeding census model (Equation 2 and Figure 1b) and the prebreeding census model (Equation 3 and Figure 1c) differ in their structures, they describe the same demographic processes (Figure 1a). Therefore, the population matrices in Equations (2) and (3) have the same dominant eigenvalue as long as the conditions of the Perron–Frobenius theorem are satisfied. Consequently, the choice between the postbreeding and prebreeding censuses is a matter of preference in age‐structured population models. These details are described in chapters 3 and 4 of Case (2000), and readers requiring more information are referred to the book.

### 2.2 Stage‐structured models

In stage‐structured models, the duration of a stage may vary among individuals. For example, eggs laid at the same time do not hatch simultaneously (Bolzan, Nava, Smaniotto, Valgas, & Garcia, 2017; Moriyama & Numata, 2008). The same principle applies to any other life stages. Consequently, individuals of a particular stage may remain in the same stage for more than one time step in stage‐structured models, in contrast to age‐structured models.

A species with two stages (juvenile and adult) was considered to illustrate the postbreeding and prebreeding censuses in stage‐structured models. When the probability of a juvenile reaching the adult stage in one time step is *γ* (assuming that the individual survives), the population matrix based on the postbreeding census is

(4) $$A_{\text{post}}=\left(\begin{array}{cc} \sigma_{J}\left(1-\gamma \right)+\sigma_{J}\gamma b & \sigma_{A}b\\ \sigma_{J}\gamma & \sigma_{A} \end{array}\right)$$

where *σ_J_* and *σ_A_* are the probability of per time step survival for a juvenile and an adult, respectively, and *b* is the number of female offspring produced by an adult. In this model, the duration of the juvenile stage is variable (denoted by a random variable *T*). In particular, the duration of the juvenile stage *T*_post_ follows a geometric distribution, and its expected duration is *E*(*T*_post_) = 1*/γ* (Caswell, 2001). In this study, subscripts “post” and “pre” in any notation describe the postbreeding and prebreeding census, respectively.

On the other hand, the population matrix with the pbreeding census is

(5) $$A_{\text{pre}}=\left(\begin{array}{cc} \sigma_{J}\left(1-\gamma \right) & \sigma_{J}b\\ \sigma_{J}\gamma & \sigma_{A} \end{array}\right)$$

in which the expected duration of the juvenile stage is *E*(*T*_pre_) = 1 + 1*/γ*. The constant (i.e., 1) is added to *E*(*T*_pre_) because the duration of an age‐0 individual (one time step) must be added to the total duration of the juvenile stage. The same principle applies in age‐structured models. In Equation (2), it is demonstrated that newborns require three time steps to reach age‐3. In Equation (3), the matrix only indicates two time steps to reach age‐3; however, a constant 1 must be added for the duration of age‐0. Because of this difference, Equations (4) and (5) assume different probability distributions for the duration of the juvenile stage. In particular, the postbreeding model assumes that the duration of the juvenile stage follows a geometric distribution. The prebreeding census model assumes that the duration of the juvenile stage follows a geometric distribution plus a constant 1. As will be described below, Equations (4) and (5) are not the only ways to model the postbreeding census and the prebreeding census but are the predominant formulations used in published studies, and thus, it is important to know potential differences in common matrix analyses (i.e., eigenvalue and elasticity analyses) resulting from the choice between the two methods.

**Figure 2:**
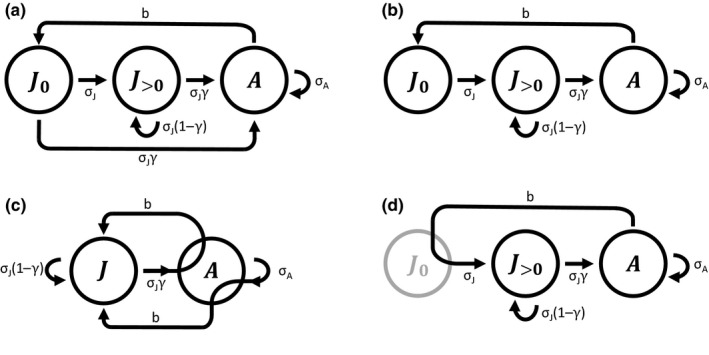
Life cycle diagrams for stage‐structured models. Nodes represent age/stage groups. *J*_0_ and *J*_>0_ represent juveniles of age‐0 and juveniles of age‐1 or older, respectively. *J* represents juveniles of all ages (i.e., *J*_0_ and *J*_>0_ combined), and *A* represents adults of all ages. (a) and (b) represent the life cycle diagrams for the postbreeding census and the prebreeding census models, respectively, without being explicit about the timing of demographic processes and censuses. It is advised to interpret these diagrams one demographic process at a time. (c) and (d) show the life cycle diagrams corresponding with the postbreeding census model (Equation 4) and the prebreeding census model (Equation 5), respectively. In (c), there are two arrows pointing toward the node *J* representing reproduction. The arrow originating from node *J* represents reproduction by age‐0 adults, and the arrow originating node *A* represents reproduction by age‐1 or older adults

The difference between the postbreeding census and prebreeding census can also be described by life cycle diagrams (Figure 2). Figure 2a,b represent demographic processes for the postbreeding census and the prebreeding census, respectively, without being explicit about their timing with respect to censuses. Unlike the age‐structured model where the postbreeding census and the prebreeding census describe the same demographic processes (Figure 1a), Figure 2a,b are not the same. The difference is that, in the postbreeding census model, it is possible for an age‐0 juvenile to become an adult in one time step (the arrow originating from *J*_0_ and pointing toward *A* in Figure 2a), whereas this transition is impossible in the prebreeding census (Figure 2b). Because of this difference, juveniles must spend at least two time steps to mature in the prebreeding census model. Life cycle diagrams that are consistent with Equation (4) (Figure 2c) and Equation (5) (Figure 2d) are also shown for references. Their formulation follows Figure 1b,c of age‐structured models. However, there are self‐directing arrows (arrows originating and returning to the same node) when it is possible for an individual to remain in the same stage for more than one time step.

## 3 RESULTS

Before describing differences in predictions between the postbreeding and prebreeding models, a related common problem in the prebreeding census‐based models is briefly discussed. As described earlier, models based on the postbreeding and prebreeding censuses assume different probability distributions for the juvenile stage. More generally, when a model is not based on juvenile and adult stages, the two methods assume different distributions for the first stage of life that results from breeding (e.g., egg and seed stages). Studies often do not recognize this detail and incorrectly parameterize population matrices especially in prebreeding census models (Birt et al., 2009; Cibils‐Stewart, Sandercock, & McCornack, 2015; Germano & Picollo, 2016; Rand, Richmond, & Dougherty, 2017). A common mistake is incorrectly assuming that *E*(*T*_pre_) = 1*/γ*, extending the expected stage duration one step longer than intended. For example, a sea turtle study (Crouse et al., 1987) initially used the prebreeding census and assumed the duration of egg stage was 2 years, although it was intended to be 1 year. However, the authors reanalyzed the data correctly using the postbreeding census (Crowder et al., 1994).

Incorrect parametrization assumes a longer than intended duration of the first stage (e.g., delayed maturation), causing an underestimation of the finite rate of increase. The significance of this difference depends on the objective and results of each study; however, it may lead to qualitative differences in population dynamics. For example, an incorrectly parameterized model may predict that a population is declining to extinction, whereas the correctly parameterized model predicts that the population is increasing. However, regardless of the extent of the differences, there is no justification for incorrectly parameterizing models. Therefore, in the following comparisons between the prebreeding census and postbreeding census models, it is assumed that the prebreeding census models are appropriately parameterized such that *E*(*T*_post_) = *E*(*T*_pre_) accomplished by setting *γ*_pre_ = *γ*_post_ */*(1 − *γ*_post_) where *γ*_pre_ and *γ*_post_ are *γ* used in the prebreeding and postbreeding models, respectively. Therefore, both models describe the same intended duration of the juvenile stage.

### 3.1 Finite rate of increase

The finite rate of increase represented by the dominant eigenvalue *λ* of **A** (*λ*_post_ for the postbreeding census model and *λ*_pre_ for the prebreeding census model) is often the main interest of matrix population studies. When *λ > *1, the population size increases in the long run, whereas when *λ < *1, the population decreases to extinction.

The finite rate of increase is compared between the two models when the model assumes an identical expected duration of the juvenile stage. The expressions of the dominant eigenvalue for Equations (4) and (5), respectively, are

(6) $$\lambda_{\text{post}}=\frac{1}{2}\left[\sigma_{J}\left(1-\gamma +\gamma b\right)+\sigma_{A}+\sqrt{{\left[\sigma_{J}\left(1-\gamma +\gamma b\right)-\sigma_{A}\right]}^{2}+4\sigma_{A}\sigma_{J}\gamma b}\right]$$

(7) $$\lambda_{\text{pre}}=\frac{1}{2}\left[\sigma_{J}\left(1-\gamma \right)+\sigma_{A}+\sqrt{{\left[\sigma_{J}\left(1-\gamma \right)-\sigma_{A}\right]}^{2}+4\sigma_{J}\gamma b}\right].$$

**Figure 3:**
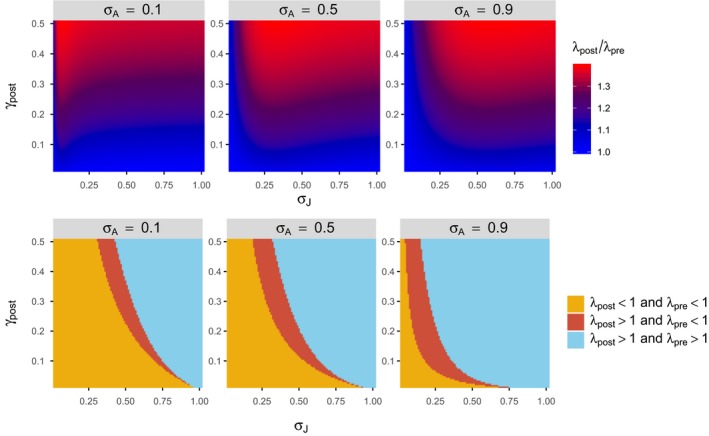
Comparison between the postbreeding and prebreeding census models when *b* = 5. *σ_J_* varies from 0.01 to 1, and *γ*_post_ varies from 0.01 to 0.5. *γ*_pre_ = *γ*_post_ */*(1 − *γ*_post_). *λ*_post_ and *λ*_pre_ are Equations (6) and (7), respectively. The top figures show the values of *λ*_post_ */λ*_pre_, and the bottom figures show the three conditions described in the figure key

*λ*_post_ is greater than *λ*_pre_ when *γ*_pre_ = *γ*_post_ */*(1 − *γ*_post_). Specific results when *b* = 5 are shown in Figure 3. The importance of this difference depends on the nature of the study. However, the difference also leads to qualitatively different predictions in population growth. In particular, when a postbreeding census model predicts that the population size increases (*λ*_post_ *> *1), the corresponding prebreeding census model predicts that the population decreases to extinction (*λ*_pre_ < 1; Figure 3).

### 3.2 Elasticity

Although the finite rate of increase *λ* is of main interest, studies often attempt to identify the relative importance of demographic parameters in determining *λ* rather than *λ* itself. In particular, the proportional change in the population growth rate for a proportional change in a demographic parameter, known as elasticity, is commonly examined (Benton & Grant, 1999; Caswell, 2001; de Kroon, Plaisier, Groenendael, & Caswell, 1986). The elasticity of *λ* to a demographic parameter *p* is defined as $e_{p}=\frac{p}{\lambda }\frac{\partial \lambda }{\partial p}$ where *p* ∈ {*σ_J_, σ_A_, γ, b*} in Equations (4) and (5). Elasticity analysis is frequently used in applied studies (e.g., conservation and pest management) in an attempt to identify an important target for application plans (Crouse et al., 1987; Shea & Kelly, 1998; Silvertown, Franco, & Menges, 1996). For example, $e_{\sigma_{J}}>e_{\sigma_{A}}$ would suggest that it is more effective to focus on protecting (in case of conservation) or killing (in case of pest management) juvenile organisms. Similarly, if the population growth rate *λ* is used as a surrogate for evolutionary fitness, elasticities may represent selection pressures acting on each life‐history parameter (Benton & Grant, 1999).

The elasticity of *λ* to each demographic parameter depends on the specific values of the four parameters (*σ_J_, σ_A_, γ*, and *b*). For Equations (4) and (5), the effects of *σ_J_* and *σ_A_* are summarized according to their ratio *σ_J_/σ_A_*. In other words, the elasticity of *λ* to each parameter depends on the three quantities (*σ_J_/σ_A_*, *γ*, and *b*) rather than the four, described in Appendix A. For elasticities, superscript is used to describe the postbreeding and prebreeding censuses (e.g., the elasticity of *λ* to *σ_J_* is $e_{\sigma_{J}}^{\text{post}}$ for the postbreeding census and $e_{\sigma_{J}}^{\text{pre}}$ for the prebreeding census).

**Figure 4:**
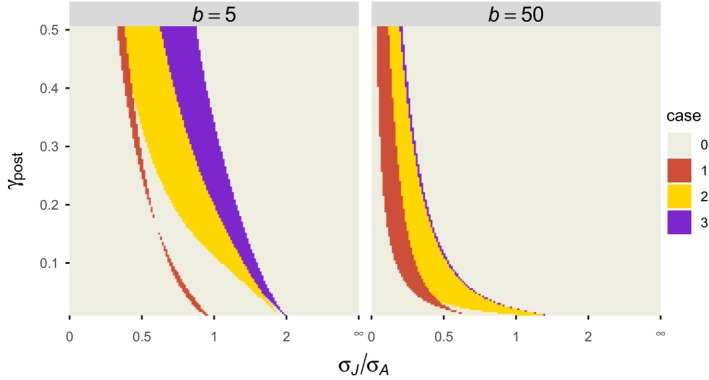
Comparison of elasticity analysis results between the postbreeding and prebreeding census models. The mathematical expressions for the elasticities of the four parameters (*σ*_J_ *, σ*_A_ *, γ*, and *b*) are shown in Appendix A. The parameter associated with the largest elasticity is rank 1, and the parameter of the second largest elasticity is rank 2, and so on. Case 1: The rank 1 parameter is different between the postbreeding and prebreeding census models. Case 2: The rank 1 parameter is identical, but the rank 2 parameter is different. Case 3: The rank 1 and rank 2 parameters are identical, but the rank 3 parameter is different. Case 0: All ranks are identical. *γ*_pre_ = *γ*_post_ */*(1 − *γ*_post_). 1 ≤ *σ_J_/σ_A_* ≤ ∞ corresponds to 1 ≥ *σ_A_/σ_J_* ≥ 0; linearly varied *σ_A_/σ_J_* was used in the computation and its reciprocal is shown as *σ_J_/σ_A_*

The results of an elasticity analysis based on the postbreeding and prebreeding censuses may be compared according to the ranking of elasticities within each model. In each model, the four parameters (*σ_J_, σ_A_, γ*, and *b*) may be ranked according to their elasticities. For example, when $e_{\sigma_{J}}^{\text{post}}>e_{\sigma_{A}}^{\text{post}}>e_{\gamma }^{\text{post}}>e_{b}^{\text{post}},$ *σ_J_* is rank 1, *σ_A_* is rank 2, etc. in the postbreeding census model. When the ranking from the postbreeding census model and the corresponding prebreeding census model is identical, the difference (i.e., Equations 4 and 5) makes no difference in the elasticity analysis when only the ranking is concerned. However, differences between the two models are readily observed, especially when the juvenile survival rate is lower than the adult survival rate (Figure 4).

## 4 DISCUSSION

It is well established that in age‐structured population models, the choice between the prebreeding and postbreeding censuses is a matter of preference (but see Cooch, Gauthier, & Rockwell, 2003). Consequently, the detail is glossed over in stage‐structured population models, even though the same equivalence is not applicable in stage‐structured models. However, as described in this study, an arbitrary choice of prebreeding census and postbreeding census may lead to contradicting conclusions in stage‐structured models. Thus, careful consideration is required when building a stage‐structured matrix population model.

This study used a specific example, two‐stage populations and geometric durations of the juvenile stage. However, the same types of differences exist regardless of the stage structure or distribution of the stage durations. For example, in a species consisting of egg, larva, pupa, and adult stages, the difference discussed in this study appears in the egg stage. More generally, the difference appears in the first stage of life. The distribution of the duration of the first stage in a prebreeding census model is generally in the form of 1 + *T*, where *T* is the distribution of the first stage described by the matrix model. This is true irrespective of the distribution of *T* such as nongeometric distributions (Caswell, 2001; Okuyama, 2018). Conventional postbreeding census models are not characterized by this constraint (i.e., the addition of a constant 1). However, both the postbreeding census and prebreeding census can be used to modify the distribution of the first stage. For example, the postbreeding census model equivalent to the prebreeding census model of Equation (5) is

(8) $$A_{\text{post}}=\left(\begin{array}{ccc} 0 & \sigma_{J}\gamma b & \sigma_{A}b\\ \sigma_{J} & \sigma_{J}\left(1-\gamma \right) & 0\\ 0 & \sigma_{J}\gamma & \sigma_{A} \end{array}\right)$$

where the juvenile stage is divided into two classes (age‐0 vs. age‐1 and older). Similarly, the prebreeding census model that is equivalent to the postbreeding census model of Equation (4) is

(9) $$A_{\text{pre}}=\left(\begin{array}{cc} \sigma_{J}\left(1-\gamma \right) & \sigma_{J}b\left(1-\gamma \right)\\ \sigma_{J}\gamma & \sigma_{A}+\sigma_{J}b\gamma \end{array}\right)$$

where the term $\sigma_{J}b\gamma$ in row 2 column 2 represents newborns that become adults in one time step. Because of the equivalence, for example, the dominant eigenvalues of Equations (5) and (8) are identical. Similarly, the dominant eigenvalues of Equations (4) and (9) are identical. However, studies that use the postbreeding census conventionally follow Equation (4) rather than Equation (8), and studies that use the prebreeding census conventionally follow Equation (5) rather than Equation (9).

**Figure 5:**
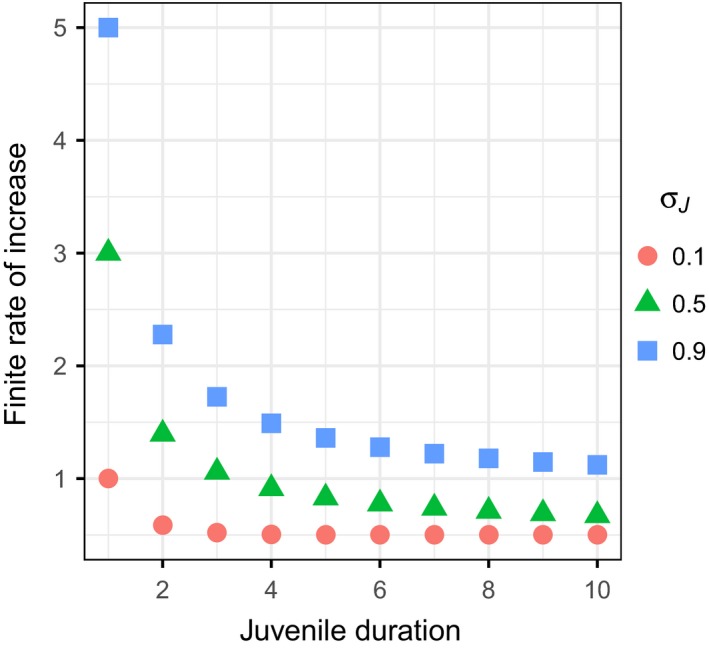
Relationship between the finite rate of increase and the duration of the juvenile stage. The duration of juvenile stage is fixed for all individuals. For example, when juvenile duration is 4, all juveniles that survived for four time steps wl become adults. *b* = 5 and *σ_A_* = 0.5

Because the only difference between the two models (Equations 4 and 5) is their assumptions of the duration of the juvenile stage, differences in model predictions (e.g., Figures 3 and 4) also arise from this difference. When *T*_post_ and *T*_pre_ are random variables describing the duration of the juvenile stage in the postbreeding and prebreeding census models, respectively, *T*_post_ follows a geometric distribution with a mean 1*/γ*_post_, whereas *T*_pre_ follows a geometric distribution with mean 1*/γ*_pre_ plus a constant 1. However, even when the means are set equal *E*(*T*_post_) = *E*(*T*_pre_), other properties of the distributions are different. For example, when *γ*_pre_ = *γ*_post_ */*(1 − *γ*_post_), Var(*T*_post_)*>*Var(*T*_pre_) where Var(·) describes variance. Another difference is the minimum duration of the juvenile stage. In Equation (4), some juveniles advance to the adult stage in one time step, whereas in Equation (5), all juveniles spend at least two time steps as juveniles. This early maturity in the postbreeding census model may explain why *λ*_post_ is generally greater than *λ*_pre_ (Figure 3). Figure 5 shows the population growth rate when the duration of the juvenile stage is fixed for all juveniles. As the duration of the juvenile stage increases for all individuals, the population growth rate (*λ*) decreases in an accelerated manner. The relationship between *λ* and the duration of the juvenile stage is convex. Because of this, even when the expected duration of the juvenile stage is identical, the greater variance in the postbreeding census offers an advantage due to Jensen's inequality (Ruel & Ayres, 1999).

Because both the postbreeding census and the prebreeding census can be used to describe the distribution of the first‐stage duration flexibly (e.g., Equations 8 and 9), the question of choosing to use the prebreeding or postbreeding census is secondary. The primary and more sensible question may be whether 1 + *T*_−0_ or *T*_+0_ is a suitable form of distribution in describing stage duration where *T*_−0_ is the distribution of the duration of the first stage excluding age‐0, while *T*_+0_ is the distribution of the duration of the first stage including age‐0. In some cases, the use of 1 + *T*_−0_ (the conventional formulation of the prebreeding census) is clearly not advisable. The first of such cases is when the expected duration of the first stage is less than two time steps because the minimum expected value of 1 + *T*_−0_ is 2. The only situation in which the conventional prebreeding census formulation may be used even when an expected duration is less than two time steps is when all individuals advance to the next stage in one time step as in Equation (3) where the first stage/age is not described in the matrix (e.g., Arnold et al., 2006). Second, even when an expected duration is longer than two time steps, the use of 1 + *T*_−0_ is not recommended when some individuals advance to the next stage in one time step. For example, in some insect species, eggs hatch within 1 day after being laid (Ekesi, Nderitu, & Rwomushana, 2006; Vargas, Walsh, Jang, Armstrong, & Kanehisa, 1996). If matrix models are constructed with the time unit of day, unless all eggs hatch within a day, the conventional formulation of the prebreeding census should not be used because it assumes that such individuals do not exist. As mentioned above, the minimum possible value is another property of probability distribution (similar to mean and variance) that may have an important influence on population growth.

As for the choice between 1 + *T*_−0_ and *T*_+0_ in other cases, there is no general answer to this problem as it is ultimately determined by the target stage duration distribution of the study organisms. In fact, it does not even make sense to focus on these two cases as we can consider 2 + *T*_−{0_ *_,_*_1}_ (where *T*_−{0_ *_,_*_1}_ is the random variable describing the stage duration excluding age‐0 and age‐1) or 3 + *T*_−{0_ *_,_*_1_ *_,_*_2}_ among many other possible descriptions of stage durations (furthermore, *T* does not have to be a geometric distribution). The distinction between 1 + *T*_−0__and_ *T*_+0_ discussed in this study simply emerges as an artifact of the conventional model structure. Yet, the distinction can qualitatively influence results, which also has important implications for comparative or meta‐analysis studies because the choice between the prebreeding census and postbreeding census is casually made without any justification in most studies. The distribution of stage duration has received considerably less attention compared with other demographic parameters such as survival and fecundity (de Valpine, Scranton, Knape, Ram, & Mills, 2014). Because demographic characteristics do not independently influence population dynamics, neglecting one detail can render an entire model unreliable even when the detail is not of primary interest. Therefore, though simple, the distinction between the prebreeding and postbreeding census models must be clearly recognized and the assumption regarding the distribution of the first stage should be actively made rather than be a passive (unintended) consequence of the choice of a modeling method.

## CONFLICT OF INTEREST

None declared.

## AUTHOR CONTRIBUTION

TO performed all work presented in this study.

## Data Availability

This study is not based on data.

## APPENDIX A

The elasticity of *λ* to a parameter *p* where *p* ∈ {*σ_J_, σ_A_, γ, b*} is shown below. The superscripts describe postbreeding census (post) or prebreeding census (pre).

(10) $$e_{\sigma_{J}}^{\text{post}}=\frac{1}{2}\left[1+\frac{1-\gamma +\gamma b-f}{\sqrt{M}}\right]$$

(11) $$e_{\sigma_{A}}^{\text{post}}=\frac{1}{2}\left[1-\frac{1-\gamma +\gamma b-f}{\sqrt{M}}\right]$$

(12) $$e_{\gamma }^{\text{post}}=\frac{\gamma }{2\left(1-\gamma \right)}\left[\frac{\left(2-\gamma \right)b+\gamma -1+f}{\sqrt{M}}-1\right]$$

(13) $$e_{b}^{\text{post}}=\frac{\gamma b}{\sqrt{M}}$$

(14) $$e_{\sigma_{J}}^{\text{pre}}=\frac{1}{2}\left[1+\frac{4\gamma b+\left(1-\gamma -f\right)\left(1-\gamma +f+\sqrt{L}\right)}{L+\left(1-\gamma +f\right)\sqrt{L}}\right]$$

(15) $$e_{\sigma_{A}}^{\text{pre}}=\frac{1}{2}\left[1-\frac{4\gamma b+\left(1-\gamma -f\right)\left(1-\gamma +f+\sqrt{L}\right)}{L+\left(1-\gamma +f\right)\sqrt{L}}\right]$$

(16) $$e_{\gamma }^{\text{pre}}=\frac{1}{2}\left[1+\frac{4f-\left(1+\gamma +f\right)\left(1-\gamma +f+\sqrt{L}\right)}{L+\left(1-\gamma +f\right)\sqrt{L}}\right]$$

(17) $$e_{b}^{\text{pre}}=\frac{2\gamma b}{L+\left(1-\gamma +f\right)\sqrt{L}}$$

where *f* = *σ_A_/σ_J_*, *M* = (1 – *γ *+ *γb* − *f*)^2^+4*fγb*, and *L* = (1 – *γ *− *f*)^2^ + 4*γb*.
